# Dosimetric comparison of VMAT standard optimization (SO) and multi‐criteria optimization (MCO) treatment plans with standard mode delivery (STD) or sliding window (SW) for head and neck cancer

**DOI:** 10.1002/acm2.14013

**Published:** 2023-05-05

**Authors:** Julien Rolland, Véronique Favrel, Pierre Fau, Hugues Mailleux, Agnès Tallet

**Affiliations:** ^1^ Department of Medical Physics Centre Hospitalier InterCommunal des Alpes du Sud Gap France; ^2^ Department of Medical Physics Institut Paoli Calmettes Marseille France; ^3^ Department of Radiotherapy Institut Paoli Calmettes Marseille France

**Keywords:** H&N, MCO, QA, sliding windows, VMAT, volumetric modulated arc therapy

## Abstract

**Purpose:**

A new development on the RayStation treatment planning system (TPS) allows a plan to be planned by imposing a constraint on the leaf sequencing: all leaves move in the same direction before moving again in the opposite direction to create a succession of sliding windows (SWs). The study aims to investigate this new leaf sequencing, coupled with standard optimization (SO) and multi‐criteria optimization (MCO) and to compare it with the standard sequencing (STD).

**Methods:**

Sixty plans were replanned for 10 head and neck cancer patients (two dose levels simultaneously SIB, 56 and 70 Gy in 35 fractions). All plans were compared, and a Wilcoxon signed‐rank test was performed. Pre‐processing QA and metrics of multileaf collimator (MLC) complexity were studied.

**Results:**

All methodologies met the dose requirements for the planning target volumes (PTVs) and organs at risk (OARs). SO demonstrates significantly best results for homogeneity index (HI), conformity index (CI), and target coverage (TC). SO‐SW gives best results for PTVs (*D*_98%_ and *D*_2%_) but the differences between techniques are less than 1%. Only the *D*_2%,PTV‐56 Gy_ is higher with both MCO methods. MCO‐STD offer the best sparing OARs (parotids, spinal cord, larynx, oral cavity). The gamma passing rates (GPRs) with 3%/3 mm criteria between the measured and calculated dose distributions are higher than 95%, slightly lowest with SW. The number of monitor units (MUs) and MLC metrics are higher in SW show a higher modulation.

**Conclusions:**

All plans are feasible for the treatment. A clear advantage of SO‐SW is that the treatment plan is more straightforward to planning by the user due to the more advanced modulation. MCO stands out for its ease of use and will allow a less experienced user to offer a better plan than in SO. In addition, MCO‐STD will reduce the dose to the OARs while maintaining good TC.

## 1 INTRODUCTION

Head and neck cancer is the sixth most common cancer in the world, causing approximately 431 000 deaths from 835 000 cases each year.^1^, ^2^

Head and neck cancer is a complicated challenge in dosimetric planning due to the irregular target volume and the proximity of many organs at risk (OARs). The irradiation of OARs can cause numerous adverse effects on the quality of life of patients such as xerostomia, dysphagia, or hearing loss.^3^, ^4^, ^5^

Planning has changed significantly in recent years, from 3D conformal radiation therapy (3DCRT) with lateral beams to intensity modulated radiation therapy (IMRT) and finally to volumetric modulated arc therapy (VMAT). The advent of IMRT and VMAT has resulted in a much more conformal and homogeneous treatment of planning target volumes (PTVs) and a significant dose reduction in OARs due to the modulation performed by the multileaf collimator (MLC). In VMAT, treatment is achieved by a combination of accelerator arm rotation and dynamic management of the MLC with continuous irradiation, and the treatment time is reduced.

New advances have appeared with the multi‐criteria optimization (MCO)^6^ and recently with the sliding window VMAT sequencing (SW) (like the sliding window in IMRT).

Optimization MCO generates a Pareto surface containing a spectrum of optimal plans, each point on the surface representing an optimal solution with different trade‐off objectives. The user can finetune treatment plans by moving sliders in real‐time to find the right balance between conflicting clinical goals. The plan remains Pareto optimal with all constraints respected—no objectives can be improved without negatively impacting others.^7^

Studies have evaluated the effectiveness of MCO compared to the standard optimization (SO) inverse planning method with a clear improvement in treatment plans.^8^, ^9^, ^10^, ^11^, ^12^

In addition, MCO allows a less experienced user of inverse planning to reduce planning time and to propose treatment plans of similar quality in complex locations such as the head and neck region (H&N).^13^, ^14^

The RayStation 8B release introduces a sliding window sequencing (SW). The standard sequencing (STD) does not impose any constraints on leaf travel. On the contrary, the sliding windows sequencing imposes one: all leaves move in the same direction, opening a slot, closing it, and then moving in the opposite direction, making a new slot, and so on for the duration of the arc. This new sequencing is available for VMAT in SO and MCO.

The aim of the study was to analyze all the possibilities offered by the RayStation planning system in VMAT technique for the treatment of the H&N: SO versus MCO, coupled with the new sequencing sliding window (SW) and the STD of the MLC.

## 2 MATERIALS AND METHODS

### 2.1 Patient selection

10 patients treated for H&N were replanned. For this study, the patients were anonymized.

### 2.2 Simulation and contour

CT data were acquired with the patient immobilized in the supine position with a 4−5 fixation point thermoplastic mask covering the head and shoulders on a CT General Electric Optima580‐RT. The CT was merged with the iv‐iodine contrast enhancement. The diagnostic MRI may be fused with the planning CT at the physician's request. The gross tumor volume (GTV), the clinical target volumes (CTVs), and the OARs were delineated using international guidelines.^15^, ^16^ The CTV‐70 Gy was generated by isotropic expansion of 1 cm around the GTV. The PTVs (PTV‐70 Gy and PTV‐56 Gy) were defined as the respective CTVs (CTV‐70 Gy and CTV‐56 Gy) plus an isotropic margin of 4 mm. The skin of 3 mm thick layer under the patient surface was excluded from the PTVs to avoid optimization problems in the build‐up zone and overdose to the skin. For the PTV‐56 Gy, we exclude the PTV‐70 Gy plus a margin of 6 mm to avoid overlapping volume in the optimization process.

### 2.3 Prescription and dose

Two dose levels are prescribed simultaneously over 35 fractions: 56 and 70 Gy. The constraints used for the validation of the plan and the considered OARs are summarized in Table 1. For the parotids, the goal is to respect the constraints for the two parotids (contralateral and ipsilateral) if and only if the coverage of the PTVs is not degraded (the CTVs must be covered by the 99% prescribed dose at the level of the parotids).

**TABLE 1:** Head and neck planning acceptance criteria.

Volumes	Constraints
PTV‐56 Gy	*D*_98%_ ≥ 95% prescribed dose, 53.2 Gy
	*D*_2%_ ≤ 107% prescribed dose, 59.92 Gy
PTV‐70 Gy	*D*_98%_ ≥ 95% prescribed dose, 66.5 Gy
	*D*_2%_ ≤ 107% prescribed dose, 73.5 Gy
Parotid glands	*D*_50%_ ≤ 30 Gy
	*D*_mean_ ≤ 26 Gy
Spinal cord	*D*_0.01cc_ ≤ 45 Gy
Brainstem	*D*_0.01cc_ ≤ 50 Gy
Brachial plexus	*D*_0.01cc_ ≤ 62 Gy
Larynx	*D*_mean_ ≤ 50 Gy *D*_0.01cc_ ≤ 70 Gy
Oral cavity minus PTV‐56 Gy	*D*_mean_ ≤ 45 Gy
Neck	*D*_mean_ : ALARA
Mandible	*D*_mean_ ≤ 60 Gy
Optic chiasma	*D*_0.01cc_ ≤ 54 Gy
Optic nerve	*D*_0.01cc_ ≤ 54 Gy
Lens	*D*_0.01cc_ ≤ 7 Gy
Total eye	*D*_mean_ ≤ 15 Gy
Pituitary gland	*D*_0.01cc_ ≤ 45−50 Gy
Cochlea	*D*_0.01cc_ ≤ 50 Gy
Temporomandibulard joint	*D*_0.01cc_ ≤ 50 Gy *D*_mean_ ≤ 40 Gy
Thyroid	*V*_50Gy_ : ALARA

Abbreviations: ALARA, as low as reasonable achievable; *D*_0.01cc_, dose to 0.01 cc of the volume; *D*, dose; Dx, dose to x% of the volume; PTV, planning target volume; Vx, % volume receiving x Gy.

The dose to all OARs had to be kept as low as possible respecting the prescription to the PTVs as mentioned in Table 1. Moreover, plans are made to achieve the lowest dose possible for all OARs (the “as low as reasonably achievable” or “ALARA” principle) by keeping a good targets coverage. H&N is a major planning challenge, where it is difficult to manage the compromise between tumors irradiation and OARs sparing. In Section 2.4, the methods used to achieve this for both optimization algorithms are described.

The maximum dose point should be in the high‐risk PTV (PTV‐70 Gy).

### 2.4 Treatment plans

The plans were generated for an Elekta Infinity accelerator (ELEKTA, Stockholm, Sweden) with the Agility MLC, 80 pairs of 5 mm thick leaf with 6 MV photons.

The treatment planning system (TPS) RayStation in version V.10A (RaySearch Laboratories AB, Stockholm, Sweden) was used.

All plans were planned by the same user to not biasing the results due to different experiences of the planners or planning differences despite the procedures in place.

For the management of the MLC and the movement of the leaves, two leaf sequencings were used. The STD does not impose any particular constraints on leaf travel. On the contrary, the sliding windows sequencing (SW) imposes one: all leaves move in the same direction, and then moving in the opposite direction, and so on for the duration of the arc.

Plans are realized as follows:

1. Two full arcs from 179° to 181° for STD (counterclockwise and clockwise).
2. One full arc from 179° to 181° for SW (counterclockwise).
3. The arcs are intended to be delivered after the daily online cone beam CT verification (CBCT in clockwise). The direction of rotation of the arcs is designed to optimize the total time of the treatment session.
4. Collimator angle was set to 15°.
5. All arcs employed a control point every 2° of gantry rotation (obligatory for SW and the same was used for STD).
6. A 3 mm dose grid was applied.

In SW, a maximum delivery time must be set, which is not the case in SO. Different tests have practiced on SW has shown a short time was not respected by the TPS (for example with a limit of 100 s, the time estimated by the TPS was 120 to 130 s). A choice of 180 s was used because this time limit is globally respected (about 6% deviation observed). In SW, a single arc is used. As the delivery time is long, about 180 s, one additional arc in SW increases the treatment time considerably: the estimated treatment time is about 260 versus 120 s in STD (two arcs). The patient being under mask, the choice of the patient's comfort is a priority except if a clinical advantage is in favor of two arcs in SW. The tests performed with two arcs did not give any dosimetric gain and even the result was less good. The manufacturer does not recommend the use of two arcs in SW either. And finally, an optimization parameter (dual arc), described in a following paragraph, is not available in SW. Therefore, only one arc is used in SW.

In terms of optimization, the two modes available in RayStation, SO and MCO were used.

In SO, in our institution, a plan is created by manually setting the objective functions weight (weight equal to 1 for OARs, and weight equal to 100 for targets). The optimization process is performed iteratively until the clinical goals are reached. After each iterative process, the weights are increase by a value between 1 and 5 for the OARs that do not meet the clinical objectives and the weights are not modified for the targets. A review of the plan is performed, and the same iterative principle is repeated while the clinical objectives are not achieved. For the organs specified in Table 1, if the objective isn't reached, the closest value will approach. Even if the objectives of Table 1 are met, this iterative protocol is performed several times in order to decrease the dose for each OAR to its lowest possible level while considering the other OARs and PTVs. Thus, we try to get closer to the best solution: sufficient coverage of the PTVs and the lowest possible dose for the OARs.

With the dual arc feature, a second arc will be created during the sequencing stage (in opposite direction, one arc in counterclockwise, second arc in clockwise). Only one set of fluence profiles are optimized for an arc selected to be optimized as a dual arc just as for the single arc case keeping more information from the fluence maps. This method conserves leaf motion by focusing one arc on the left side of the target and the other on the right side at a given control point angle, reducing leaf openings over the OAR and increasing sparing. The dual arc feature is available for SO‐STD and MCO‐STD (not possible for SW).

The differences between SO and MCO optimization in RayStation were explained by Ghandour et al.^9^ SO uses a direct machine parameter optimization (DMPO) algorithm. A coarse arc segmentation is performed using 24° gantry spacing while converting to optimized fluence maps per initial angle. The maps are then converted into a user determined 2−4 control points per initial angle. All control points are processed to comply with the motion constraints of VMAT: max leaf speed, valid dose rates, and delivery time. This often means that leaves are shifted, and a sorting algorithm determines where to put each control point to minimize overall leaf travel for the arc. Through the creation of a Pareto database and a navigation procedure that seamlessly interpolates between the plans in the database, MCO gives the user the ability to create a final plan by considering many criteria. No criterion can be enhanced for a Pareto ideal design without compromising another criterion. In RayStation, for a plan with *n* objectives, a minimum of 2*n* Pareto plans is required to provide a workable approximation of the Pareto surface for photon optimization in RayStation. In our institute, 4*n* plans in a closer approach to the true Pareto surface are used. Constraints are utilized in addition to objectives to narrow the focus on clinically practical plans, resulting in a Pareto surface with a narrower range but higher resolution. Navigating on the Pareto surface with the navigational sliders yields an MCO plan, this plan names navigation plan. To prevent degradation while navigating other sliders in desired directions, a specific slider can be clamped to limit the range of navigation on the Pareto surface. With the navigation plan, we make sure to find the best compromise between the coverage of PTVs and the lowest dose to all OARs. Once the navigation plan is found, the fluence pattern of the selected Pareto optimal plan is converted to deliverable machine parameters for each control point and the final dose is calculated. This final deliverable plan is used for clinical evaluation.

A convex Pareto surface approximation for fluence maps, a fluence map optimization for the discrete Pareto surface representation (navigational best plan), and DMPO VMAT optimization to generate MLC segments are the three distinct algorithms used by MCO‐VMAT. To save calculation time, both VMAT optimization algorithms used a single‐value pencil beam kernel decomposition for approximation. To reduce differences between the pencil beam and collapsed cone algorithms (used in the final dosage calculation), an intermediate dose is used. The final plan in dose has a slight difference to the navigation plan, due to the conversion to dose. The method with the standard MLC sequencing is named MCO‐STD.

The RayStation 8B release introduces a segment‐based optimization mode for VMAT, using sliding window sequencing, where Pareto plans are generated by DMPO. The deliverable plan is created by control point interpolation, resulting in a high level of agreement between the navigated dose and the dose of the deliverable plan, this method names MCO‐SW.

One recommendation of the manufacturer is to limit the number of monitor units (MUs) with the SW. Indeed, the plans made in SW give much higher numbers of MUs to deliver the same dose as our reference methods SO‐STD and MCO‐STD. Without applying a limit (no limit, nl), the number of MUs is superior at 1000. Three plans were planned with a constraint on the maximum number of MUs (400, 600, and 800) to study the influence on the modulation of the MLC and to visualize or not a dosimetric impact. At 400 MUs, the generated plans do not respect the dosimetric constraints of Table 1 and are not included in this study. With the STD sequencing, whether in SO or MCO, the number of MUs is not excessive and no limit is used. In MCO‐SW, the number of MUs increases too. The results of the plans with a limit on the number of MUs are not proposed because the generated plans don't respect the dosimetric constraints Table 1. Constraints at 600 and 800 MUs are used and an unconstrained plan (no limit, nl), respectively named SO‐SW800, SO‐SW600, and SO‐SWnl.

The six configurations evaluated were: SO‐STD, MCO‐STD, SO‐SWnl, SO‐SW600, SO‐SW800, and MCO‐SW.

### 2.5 Comparison of plans

For all patients, target coverage (TC), homogeneity index (HI), and conformity index (CI) for PTVs were assessed, as well as dose or coverage values *D*_98%_, *D*_2%_, and *D*_50%_.

The homogeneity index (HI)^17^ is defined as follows:

(1) $$\mathrm{HI}\;=\frac{D_{2\%}-D_{98\%}}{D_{50\%}}$$

where *D*_2%_ represents the maximum dose delivered in 2% of the PTV volume and *D*_98%_ is the minimum dose delivered in 98% of the PTV volume. *D*_50%_ is the median dose of the PTV. HI is an index that represents the homogeneity of the dose in the PTV and a value of 0 is optimal.

According to the CI defined by Paddick,^18^ the CI is defined as follows:

(2) $$\mathrm{CI}\;=\frac{V_{T_{\mathrm{pres}}}^{2}}{\mathrm{TV}\times V_{\mathrm{pres}}}$$

where $V_{T_{\mathrm{pres}}}$ is the volume of the PTV that receives a dose equal to or greater than the prescribed dose. TV is the volume of the PTV. *V*_pres_ is the volume that receives a dose equal to or greater than the prescribed dose. The CI value is between 0 and 1, the optimal value is 1.

The target coverage index TC is defined as follows:

(3) $$\mathrm{TC}\;=\frac{V_{T_{\mathrm{pres}}}}{\mathrm{TV}}$$

where TC is the fraction of the PTV that receives at least the prescribed dose. For perfect coverage, TC is equal to 1.

The volumes of the isodose receiving 50% of the prescribed dose of each PTV (28 and 35 Gy isodoses) were analyzed.

For the OARs, the values corresponding to Table 1 were reported.

### 2.6 Quality control

Before treatment, dosimetric verifications are performed following the recommendations made by Miften et al.^19^ for all routine VMAT QA. The measurements were performed using an ArcCheck system (SunNuclear Corporation, Melbourne, FL) diode array.

Gamma index analysis was performed at the 3%/3 mm dose difference and distance to agreement criteria, with a 10% low dose threshold and global dose normalization. Gamma passing rate (GPR) of 95% is used as a tolerance criterion to identify plans that may be more susceptible to dose mismatches between measurement and TPS calculation. Plans are considered to pass if the GPRs are higher than the tolerance level, whereas they are considered to fail if the GPRs are lower.

In addition, a GPR with local normalization was performed because it is more stringent than global normalization to visualize more fines differences between different plans.

All plans were measured in a row, on the same day, to avoid uncertainty due to variations of phantom setup and the accelerator (MLC calibration mainly).

### 2.7 MLC complexity metrics

The complexity metrics selected for this study were used to describe the general MLC movement and aperture shape of treatments. The following metrics were considered:

1. MU: the number total of MUs
2. the *M* metric (in mm^−1^) introduced by Young et al.^20^, ^21^ This metric is calculated from the MLC opening per control point, per arc, and per plane. It is defined by:
(4) $$M\;=\;\frac{1}{\mathrm{MU}}\sum_{i\;=\;1}^{N}MU_{i}\times \frac{y_{i}}{A_{i}}$$
  where the sum is over all control point apertures from i=1 to i=N, MU is the total number of MU in the plan, $MU_{i}\;$is the number of MU delivered through the aperture *i*, $A_{i}$ is the open area of aperture *i*, and $y_{i}$ is the aperture perimeter excluding the MLC leaf ends. *M* increase when modulation increase.
3. the modulation complexity score applied to VMAT (MCSv). To quantify the complexity of control point (CP) and in fact the complexity total of the arc, the modulation complexity score originally defined by McNiven et al.^22^ for IMRT was used. In VMAT, this MCS was adapted by Masi et al.^23^ with the MCSv. The MCSv is based on a combination of the mean value between the adjacent CP of the aperture area variability (AAV) and leaf sequence variability (LSV) weighted by the relative MU delivered between two consecutives CPs and the summed over all CPs in the arc.

The MCSv is defined as follows:

(5) $$\begin{array}{ccc} \mathrm{MCSv} & = & \sum_{i=1}^{I-1}\left[\frac{\mathrm{AA}V_{CP_{i}+}\mathrm{AA}V_{CP_{i+1}}}{2}\times \frac{\mathrm{LS}V_{CP_{i}+}\mathrm{LS}V_{CP_{i+1}}}{2}\right.\\ & & \left.\times \;\frac{MU_{CP_{i,i+1}}}{MU_{\mathrm{arc}}}\right] \end{array}$$

The MCSv is defined to indicate more complex plans with lower values. The MCSv has a value in the range from 0 to 1. No modulation corresponding at a value of MCSv equal to 1 and for example by an arc with a fixed rectangular aperture with no leaves moving during the arc. When modulation increases, the MCSv decreases.

### 2.8 Statistical analysis

Statistical analysis was performed using a Wilcoxon signed‐rank test to determine whether there were significant differences in the dose values examined. A *p*‐value smaller than 0.05 was defined as statistically significant. With this statistical method, the choice was to compare method by method to determine the potential of each one compared to the others.

## 3 RESULTS

**FIGURE 1:**
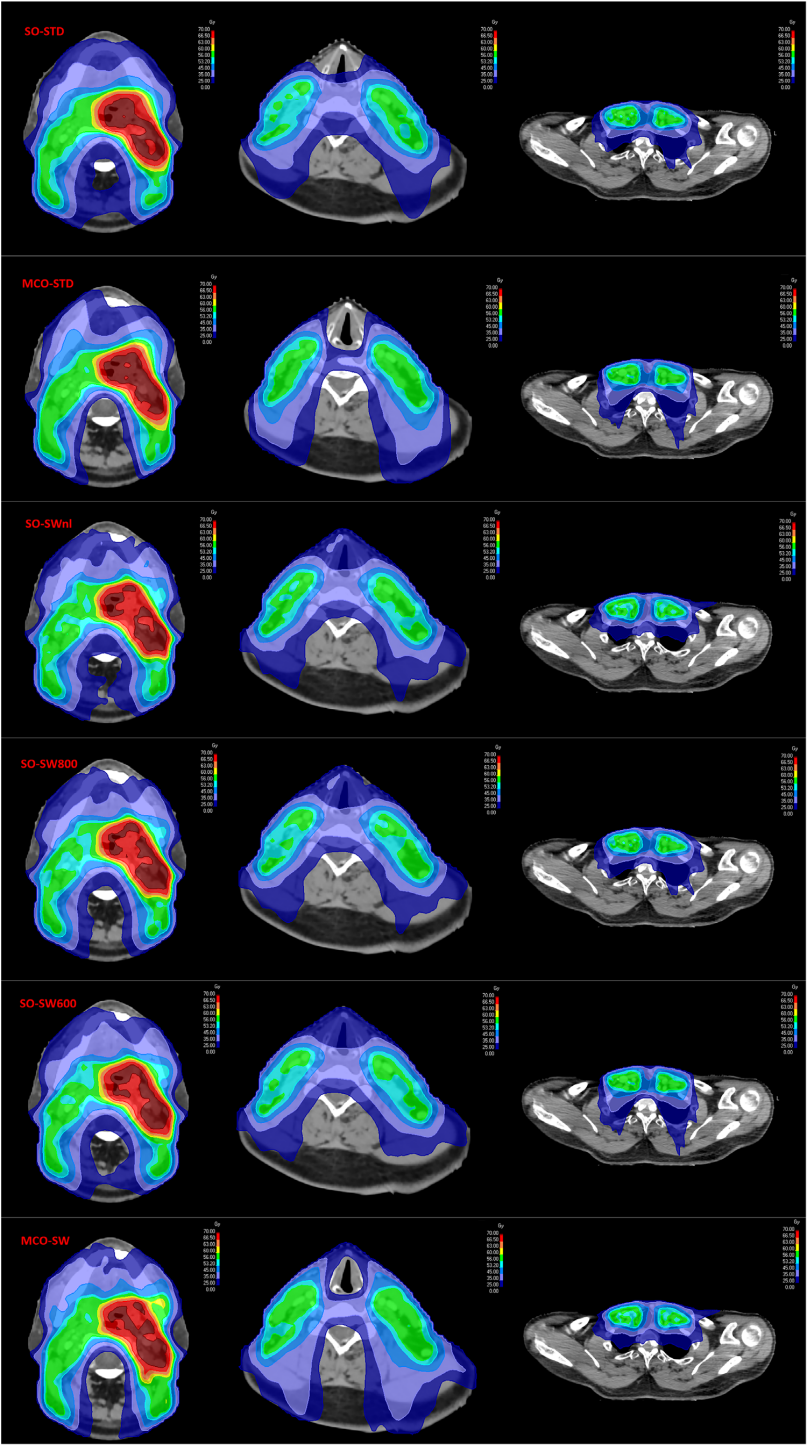
The typical dose distributions in 3 axial plans for one patient and all methodologies. Isodoses colorwash: 25, 35, 45, 53.2, 56, 60, 63, 66.5, and 70 Gy.

Figure 1 shows the typical dose distributions of all the techniques in three transversal slices for a patient.

**For the PTVs**, the dosimetric results are referenced in Table 2. A color scale is applied to give a visual aspect to the results with a gradient of red, yellow, and green colors from the worst value to the best value. A summary table of all *p*‐values to compare all dosimetric data is given in Table 3. All coverage values are respected. As shown in Table 2, results are better if the color are green. Green is dominant in the SO‐STD/SW methods as well as the intermediate values (in yellow). The worst values, in orange/red, are more represented for the MCO. The MCO‐SW is red except for the *D*_98%_ value (best value). On the *D*_98%_ and *D*_2%_ values, we note differences of less than 1% between the minimum and maximum values. Only *D*_2%_ of PTV‐56 Gy shows a dose increase for MCO of 1.5% with MCO‐STD and 2.5% for MCO‐SW compared to SO (SO‐STD and SO‐SW). For PTV‐56 Gy, SO demonstrates statically significant a lowest HI and a highest CI and TC (Tables 2 and 3) compared to MCO. A statistically significant lower HI is created by SO compared to MCO. These results are generally statistically significant in favor of the SW as shown in Table 3 where the colors (orange, yellow, and green) related to the SW methods (STD and SW) are largely dominant, on the contrary the colors (blue and purple) of both MCO, are not very frequent.

**TABLE 2:** Summary of the quantitative analysis of the dose volume histograms of target volumes for all techniques.

	SO‐STD		MCO‐STD		SO‐SWnl		SO‐SW800		SO‐SW600		MCO‐SW	
	*D*_mean_ (Gy)	SD	*D*_mean_ (Gy)	SD	*D*_mean_ (Gy)	SD	*D*_mean_ (Gy)	SD	*D*_mean_ (Gy)	SD	*D*_mean_ (Gy)	SD
*D*_98%_ PTV‐56 Gy	53.41	0.23	53.60	0.43	53.70	0.37	53.62	0.39	53.54	0.39	53.76	0.39
*D*_50%_ PTV‐56 Gy	56.02	0.18	56.49	0.19	56.05	0.04	56.04	0.05	56.06	0.09	56.83	0.39
*D*_2%_ PTV‐56 Gy	58.06	0.29	58.95	0.52	58.11	0.30	58.07	0.29	58.03	0.26	59.54	0.74
TC PTV‐56 Gy	0.76	0.03	0.73	0.05	0.77	0.03	0.77	0.04	0.76	0.04	0.73	0.03
CI PTV‐56 Gy	0.75	0.03	0.72	0.05	0.76	0.03	0.75	0.03	0.75	0.03	0.71	0.03
HI PTV‐56 Gy	0.08	0.01	0.09	0.01	0.08	0.01	0.08	0.01	0.08	0.01	0.10	0.02
*D*_98%_ PTV‐70 Gy	67.09	0.56	66.87	0.36	67.33	0.65	67.28	0.68	67.15	0.54	66.71	0.39
*D*_50%_ PTV‐70 Gy	70.12	0.12	70.12	0.13	70.04	0.04	70.06	0.04	70.11	0.08	70.03	0.23
*D*_2%_ PTV‐70 Gy	71.52	0.25	71.63	0.24	71.60	0.32	71.55	0.29	71.57	0.33	71.98	0.23
TC PTV‐70 Gy	0.84	0.05	0.82	0.03	0.84	0.05	0.84	0.04	0.84	0.04	0.83	0.04
CI PTV‐70 Gy	0.83	0.04	0.81	0.03	0.83	0.04	0.82	0.04	0.83	0.04	0.80	0.05
HI PTV‐70 Gy	0.06	0.01	0.07	0.01	0.06	0.01	0.06	0.01	0.06	0.01	0.08	0.01

*Note*: Color scale: Green to red for the best value to the worst value.

Abbreviations: CI, conformity index; HI, homogeneity index; MCO, multi‐criteria optimization; SD, standard deviation; SO, standard optimization; STD, standard sequencing; SW, sliding windows sequencing; TC, target coverture.

For a ranking for targets planning, SO‐SWnl comes first, SO‐SW800 in second, SO‐STD and SO‐SW600 in third, MCO‐STD fourth, and MCO‐SW last.

**For the OARs**, the dosimetric results are referenced in Table 4. A color scale is applied to give a visual aspect to the results with a gradient of red, yellow, and green colors from the worst value to the best value. A summary table of all *p*‐values to compare all dosimetric data is given in Table 5. The results on some OARs (eyes, lens, optic nerves, and optic chiasma) are not commented because the doses received by these organs are of the order of 2 Gy for all modalities (These structures receive only the scattered radiation. The results are given for information in Table 4). Table 5 shows a dominant color in blue corresponding to the MCO‐STD. Compared to all SO, the MCO‐STD allows a clear and statistically significant reduction of the dose to the OARs. At the level of average doses (parotids, larynx, neck, oral cavity), MCO‐STD allows to decrease the mean dose (*p* < 0.05) by approximately 4.5% to 5.0% for the parotids, 9.4% to 24.6% for the larynx, 10.9% to 13.2% for the oral cavity and 26.2 to 37.2% for the neck compared for the other plans. At the maximum dose level (*p* < 0.05) gains are 28.1% to 31.8% for the spinal cord and 39.6% to 45.5% for the brainstem compared to SO. Between MCO‐SW and MCO‐STD, the differences for the spinal cord and brainstem are respectively 1.3% and 14.1%, in favor of MCO‐STD. For the other OARs, the observation is less obvious in Table 4. There are differences but they are much less marked, and the dose values are much lower than the imposed constraints. For the pituitary gland, MCO slightly reduces the dose, but the maximum median dose delivered is less than 5 Gy for all methods. For the temporomandibular joint and cochlea (right or left), SO‐STD gets the lowest doses before MCO‐STD. SO‐SW delivers more dose to these structures as the number of MUs increases. In terms of doses outside the PTVs, that is, 50% isodoses (values in Table 6), the lowest values are obtained with SO compared to MCO with a slight advantage for SO‐SW. SO‐SW allows a gain of 57 cm^3^ and 24 cm^3^, 108 cm^3^ and 105 cm^3^, 225 cm^3^, and 147 cm^3^ respectively on isodoses 28 and 35 Gy compared to SO‐STD, MCO‐STD and MCO‐SW, statistically significant between SO‐SW and both MCO. The maximum dose point is well located in the PTV70Gy for all plans. For the OARs, the ranking proposed is as follows: MCO‐STD far superior then MCO‐SW, and SO (STD and SW).

**TABLE 3:** The statistically significant differences between the six techniques (*p*‐value, Wilcoxon signed‐rank) for the PTVs and plan data

PTVs	**SO‐SWnl** vs MCO‐SW	**SO‐SWnl** vs MCO‐STD	**SO‐SWnl** vs SO‐STD	**SO‐SWnl** vs SO‐SW600	**SO‐SW800** vs MCO‐SW	**SO‐SW800** vs MCO‐STD	**SO‐SW800** vs SO‐SW600	**SO‐SW600** vs MCO‐STD	**SO‐SW600** vs MCO‐SW	**SO‐STD** vs MCO‐STD	**SO‐ST**D vs MCO‐SW	SO‐SWnl vs SO‐SW800	SO‐STD vs SO‐SW600	SO‐STD vs SO‐SW800	**MCO‐STD** vs MCO‐SW
D_98%_ PTV‐56Gy			0.020	0.044					0.049	0.028	0.049				
D_2%_ PTV‐56GY	0.006	0.002			0.002	0.002		0.002	0.002	0.002	0.002				0.027
TC PTV‐56Gy	0.006	0.014		0.023	0.009	0.014		0.024	0.002		0.015				
CI PTV‐56Gy	0.002	0.002		0.006	0.004	0.006	0.049	0.006	0.006	0.020	0.010				
HI PTV‐56Gy	0.009	0.020			0.014	0.032		0.008	0.009	0.042	0.042				
D_98%_ PTV‐70Gy	0.027	0.037	0.033					0.049	0.018		0.044				
D_2%_ PTV‐70Gy	0.024				0.014				0.014		0.025				0.032
TC PTV‐70Gy															
CI PTV‐70Gy		0.049							0.020						
HI PTV‐70Gy	0.021	0.034			0.034	0.035			0.033	0.037	0.029				
**Plan data**	SO‐STD vs MCO‐STD	**SO‐STD** vs MCO‐SW	**SO‐STD** vs SO‐SWnl	**MCO‐STD** vs SO‐SWnl	**MCO‐STD** vs MCO‐SW	SO‐STD vs SO‐SW800	MCO‐STD vs SO‐SW800	SO‐STD vs SO‐SW600	MCO‐STD vs SO‐SW600	**SO‐SW600** vs SO‐SWnl	SO‐SW600 vs SO‐SW800	SO‐SW600 vs MCO‐SW	SO‐SW800 vs MCO‐SW	SO‐SWnl vs SO‐SW800	SO‐SWnl vs MCO‐SW
MUs	0.037	0.002	0.002	0.002	0.002	0.002	0.002	0.002	0.002	0.002	0.002	0.002	0.002	0.002	0.002
M (mm^‐1^)		0.002	0.006	0.002	0.006	0.004	0.006			0.009	0.014	0.004	0.009	0.009	
MCSv		0.002	0.006	0.006	0.006	0.002	0.002	0.002	0.002	0.006	0.006	0.006	0.006	0.002	
GPR local (3%/3mm TH = 10)		0.027	0.020	0.002	0.047					0.049					
GPR global (3%/3mm TH = 10)				0.025											

Abbreviation: GPR, gamma passing rate.

*Notes*: A *p*‐value of less than 0.05 is considered to be significant; the best method for all PTVs and plan data between the comparison is marked in gras and each with its own color highlighting the technique doing the best in the comparison; red = SO‐STD; orange = SO‐SWnl; yellow = SO‐SW800; green = SO‐SW600; blue = MCO‐STD; purple = MCO‐SW.

**FIGURE 2:**
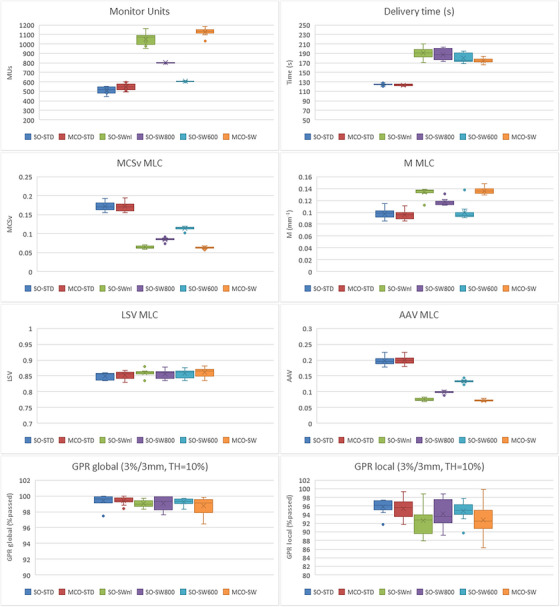
Results of monitor units, delivery time in s, complexity metrics with *M* in mm^−1^, MCSv with its components leaf sequence variability (LSV), aperture area variability (AAV), and QA pre‐treatment. GPR, gamma passing rate; MU, monitor unit.

**FIGURE 3:**
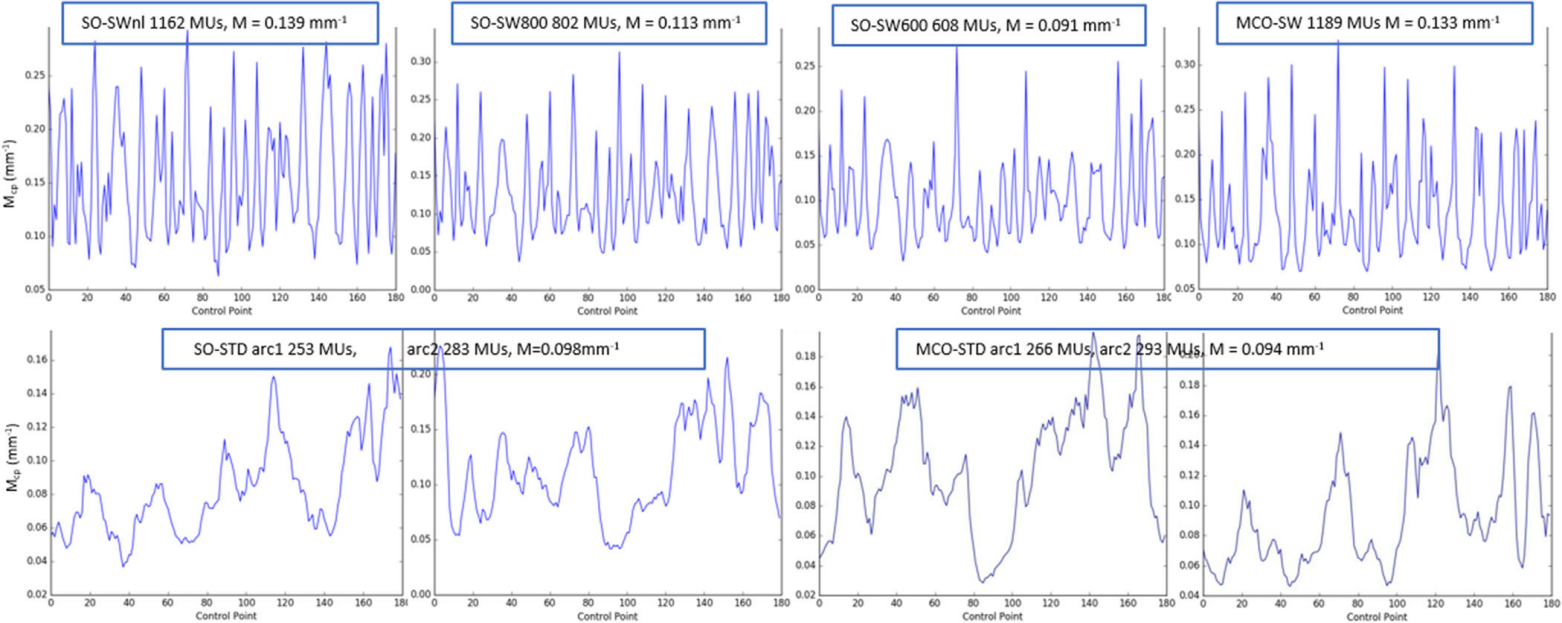
Complexity metric *M* per control point (CP) for a case.

**FIGURE 4:**
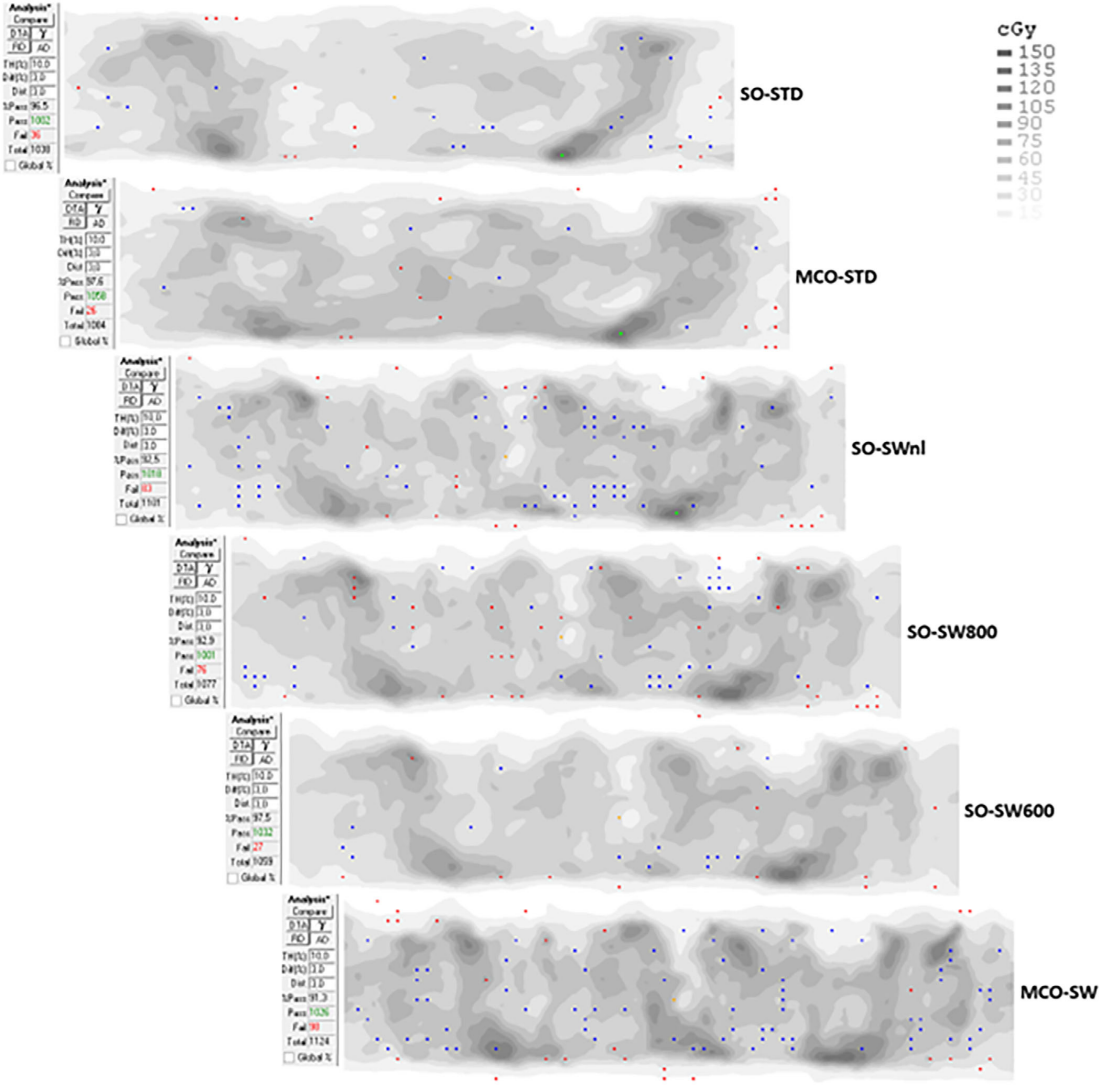
Example of a local gamma analysis 3%/3 mm. AD, absolute dose, Diff(%) criterion in dose; Dist, criterion in distance to agreement; Fail, number of points failing criteria; %Pass, percentage of evaluated measurement points passing criteria; Pass, number of points passing criteria; TH(%), threshold; Total, measurement points within threshold. Red or blue points: measured dose values above or below treatment planning system (TPS).

**Treatment** **plan data**: The number of MUs, MLC metrics, QA result, and delivery time are given in Figure 2 and are commented in this order. A summary table of all *p*‐values to compare all dosimetric data is given in Table 3. A significant increase is observed for the average MUs for MCO‐STD (548 ± 34), SO‐SWnl (1051 ± 59), SO‐SW800 (802 ± 2), SO‐SW600 (606 ± 3) and MCO‐SW (1129 ± 40), when compared to SO‐STD (514 ± 34). The increase was 7%, 105%, 56%, 18%, and 120% respectively (*p* < 0.05 for all). For the MLC metrics (M and MCSv), the modulation values are the same for the standard delivery STD: −2.2% and −0.2%, respectively when compared MCO‐STD to SO‐STD (*p* > 0.05). However, for SW, the MCSv are a lower value corresponding to a much higher modulation, statistically significant when compared with SO‐STD: −62.7% for SW‐SWnl, −63.5% for MCO‐SW. Lower modulation is found for the two sub‐techniques SO‐SW800 and SO‐SW600 compared to SO‐SWnl, where the MUs are limited during the optimization but the differences with SO‐STD are significant: −50.7% and −33.5% respectively (*p* < 0.05). For the MCSv, it is the aperture component of the MLC (AAV) that is strongly reduced in SW. For the metric M, the same phenomenon is observed, *M* decrease with the MUs for SW: 0.134, 0.117, and 0.100 mm^−1^ for SO‐SWnl, SO‐SW800, and SO‐SW600 respectively compared to 0.098 mm^−1^ for SO‐STD. Results are statistically significant. An example of the metric *M* is given by Figure 3 for a case. For the criteria 3%/3 mm and global normalization, all plans investigated have GPRs above the tolerance limit of 95% indicating all plans would be realizable for treatment. The quality control results show homogeneous results in global normalization. The differences on the mean GPRs between the methods are less than 1.0% compared to SO‐STD: 0.0%, 0.4%, 0.1%, and 0.7% for MCO‐STD, SO‐SWnl&800, SO‐SW600, and MCO‐SW respectively. For the criteria 3%/3 mm and local normalization, more restrictive, seven plans have a GPRs below 90%. These plans have a SW delivery whose five where the MUs are not limited. As the number of MUs increases, GPR deteriorates. The measurements are less good for the SW plans. The differences with the SO‐STD are 3.4%, 2.0%, and 1.2% for respectively the plans SO‐SWnl, SO‐SW800, and SO‐SW600. The results between SO‐STD and MCO‐STD are close and there are not significant differences. An example of the result for the same case is given by Figure 4. In terms of processing time, although the SW technique uses only one arc versus two arcs for STD, the delivery time is extended from 50 to 70 s (out of a time of about 125 s in STD).

**TABLE 4:** Summary of the quantitative analysis of the dose volume histograms of organs at risk for all techniques

	SO‐STD		MCO‐STD		SO‐SWnl		SO‐SW800		SO‐SW600		MCO‐SW	
	*D*_mean_ (Gy)	SD	*D*_mean_ (Gy)	SD	*D*_mean_ (Gy)	SD	*D*_mean_ (Gy)	SD	*D*_mean_ (Gy)	SD	*D*_mean_ (Gy)	SD
*D*_mean_ parotid (controlateral)	25.09	1.53	24.03	2.09	25.22	1.35	25.14	1.47	25.25	1.42	25.04	2.20
*D*_50%_ parotid (controlateral)	19.76	2.71	19.06	2.26	19.61	1.86	19.71	2.25	20.35	1.94	20.88	3.66
*D*_mean_ parotid (ipsilateral)	25.65	2.04	24.54	2.91	25.69	1.85	25.43	1.84	25.76	1.95	25.79	2.69
*D*_50%_ parotid (ipsilateral)	20.01	2.80	19.35	2.92	19.22	2.18	19.01	2.23	20.52	2.27	19.90	2.52
*D*_mean_ parotids	25.37	1.69	24.29	2.16	25.46	1.52	25.28	1.60	25.51	1.58	25.41	2.29
*D*_50%_ parotids	19.88	2.40	19.20	2.32	19.42	1.76	19.36	2.02	20.43	1.83	20.39	2.87
*D*_0.01cc_ spinal cord	38.93	2.65	30.26	3.10	38.46	1.85	38.75	2.28	39.89	1.95	30.67	2.05
*D*_0.01cc_ brainstem	37.81	5.13	25.98	7.59	37.15	5.16	36.90	5.27	36.27	4.94	29.64	8.58
*D*_mean_ larynx	44.41	6.83	35.85	10.92	44.33	6.14	43.90	6.70	44.69	6.85	39.24	10.24
*D*_0.01cc_ larynx	67.30	6.42	67.11	6.64	67.27	6.71	67.05	6.80	67.34	6.56	66.89	6.89
*D*_mean_ oral cavity‐PTV56	40.04	11.60	35.65	11.13	39.63	11.53	39.69	11.66	40.37	12.30	39.55	11.87
*D*_0.01cc_ plexus brachial	58.58	1.85	57.92	1.87	58.38	2.22	58.45	2.41	58.85	2.39	58.54	1.83
*D*_mean_ neck	19.78	3.14	14.43	1.54	19.74	1.88	19.80	2.68	19.41	1.74	18.22	1.85
*D*_mean_ mandible	41.45	11.05	42.60	11.03	41.74	10.91	41.66	11.27	41.46	11.64	42.97	10.69
*D*_0.01cc_ cochlea (right)	16.56	13.91	17.05	15.17	19.23	13.95	18.74	15.37	17.08	14.91	18.18	15.84
*D*_0.01cc_ cochlea (left)	14.48	10.18	14.79	11.58	18.38	10.52	20.25	13.93	16.56	10.76	16.56	12.15
*D*_mean_ temporomandibular joint (right)	15.57	10.69	15.79	11.01	17.35	10.39	16.49	10.43	15.93	10.24	17.17	11.32
*D*_0.01cc_ temporomandibular joint (right)	30.77	20.19	31.09	20.77	33.33	18.42	32.01	19.21	31.16	19.05	32.14	19.18
*D*_mean_ temporomandibular joint (left)	14.49	11.83	15.40	12.37	16.82	12.41	16.00	12.72	15.34	12.60	16.43	13.08
*D*_0.01cc_ temporomandibular joint (left)	26.86	19.09	29.22	17.58	30.40	16.69	28.81	17.71	27.89	17.67	29.55	18.44
*D*_mean_ pituitary gland	3.80	2.98	3.26	2.11	4.91	4.32	4.15	3.21	4.48	4.22	4.32	3.58
*V*_50Gy_ thyroid	63.05	25.37	63.55	25.21	62.26	25.54	62.41	26.10	63.22	25.82	65.64	24.68
*D*_0.01cc_ optic chiasma	2.02	1.18	1.92	0.99	2.16	1.27	2.09	1.15	2.19	1.37	2.04	1.11
*D*_0.01cc_ lens (right)	1.52	0.85	1.47	0.76	1.61	0.92	1.56	0.84	1.56	0.84	1.57	0.84
*D*_0.01cc_ lens (left)	1.53	0.92	1.48	0.79	1.66	0.98	1.60	0.90	1.61	0.94	1.62	0.91
*D*_0.01cc_ eye (right)	1.41	0.92	1.33	0.77	1.51	0.96	1.46	0.90	1.49	0.95	1.47	0.89
*D*_0.01cc_ eye (left)	1.35	0.77	1.31	0.67	1.40	0.78	1.36	0.73	1.38	0.75	1.35	0.71
*D*_0.01cc_ optic nerve (right)	2.09	1.28	2.00	1.13	2.18	1.36	2.13	1.25	2.22	1.43	2.09	1.23
*D*_0.01cc_ optic nerve (left)	2.12	1.33	2.04	1.19	2.28	1.44	2.20	1.31	2.28	1.45	2.19	1.31

**TABLE 5:** The statistically significant differences between the six techniques (*p*‐value, Wilcoxon signed‐rank) for the OARs

OARs	**MCO‐STD** vs. SO‐STD	**MCO‐STD** vs. SO‐SW800	**MCO‐STD** vs. SO‐SW600	**MCO‐STD** vs. SO‐SW	**MCO‐STD** vs. MCO‐SW	**MCO‐SW** vs. SO‐STD	**MCO‐SW** vs. SO‐SW	**MCO‐SW** vs. SO‐SW800	MCO‐SW vs. SO‐SW600	**SO‐STD** vs. SO‐SW600	**SO‐STD** vs. SO‐SWnl	**SO‐SWnl** vs. SO‐SW600	**SO‐SW80**0 vs. SO‐SW600	SO‐SWnl vs. SO‐SW800	SO‐STD vs. SO‐SW800
*D*_mean_ parotid (controlateral)	0.049			0.027	0.020										
*D*_50%_ parotid (controlateral)												0.049	0.020		
*D*_mean_ parotid (ipsilateral)	0.010			0.014	0.037										
*D*_50%_ parotid (ipsilateral)					0.014										
*D*_mean_ both parotids	0.010		0.049	0.014	0.014										
*D*_50%_ both parotids												0.049	0.014		
*D*_0,01cc_ spinal cord	0.006	0.002	0.002	0.006		0.010	0.004	0.004	0.002			0.006	0.020		
*D*_0,01cc_ brainstem	0.004	0.004	0.004	0.004		0.008	0.012	0.021	0.012	0.004					
*D*_mean_ larynx	0.016	0.016	0.016	0.016		0.031	0.047		0.016				0.047		
*D*_0,01cc_ larynx															
*D*_mean_ oral cavity‐PTV56	0.016	0.008	0.016	0.008	0.008										
*D*_0,01cc_ plexus brachial			0.049									0.027	0.014		
*D*_mean_ neck	0.002	0.002	0.002	0.006	0.002										
*D*_mean_ mandible						0.010	0.006	0.037	0.027						
*D*_0.01cc_ cochlea (right)				0.020							0.049	0.014	0.037		
*D*_0.01cc_ cochlea (left)		0.049													
*D*_mean_ temporomandibular joint (right)												0.006			
*D*_0.01cc_ temporomandibular joint (right)									0.002		0.037	0.013			
*D*_mean_ temporomandibular joint (left)				0.019		0.019			0.049		0.019	0.002			
*D*_0.01cc_ temporomandibular joint (left)												0.019			
*D*_mean_ pituitary gland		0.013		0.006	0.037	0.037	0.006				0.006			0.028	
*V*_50Gy_ thyroid				0.032			0.032								
Isodose 28 Gy (50% PTV‐56 Gy)		0.004	0.010	0.002		0.027	0.002	0.002	0.002				0.002		
Isodose 35 Gy (50% PTV‐70 Gy)				0.020		0.037	0.002	0.010	0.002						

*Note*: A *p*‐value of less than 0.05 is considered to be significant, the best method for all OARs between the comparison is marked in gras and with its own color highlighting the technique doing the best in the comparison; red = SO‐STD; orange = SO‐SWnl; yellow = SO‐SW800; green = SO‐SW600; blue = MCO‐STD; purple = MCO‐SW.

**TABLE 6:** Results for the volumes of the patient receiving 28 and 35 Gy.

	SO‐STD		MCO‐STD		SO‐SWnl		SO‐SW800		SO‐SW600		MCO‐SW	
	Volume (cm^3^)	SD	Volume (cm^3^)	SD	Volume (cm^3^)	SD	Volume (cm^3^)	SD	Volume (cm^3^)	SD	Volume (cm^3^)	SD
Isodose 28 Gy	2369.8	407.2	2420.7	387.0	2313.1	389.6	2343.0	372.2	2312.3	378.4	2538.2	411.0
Isodose 35 Gy	1845.7	299.6	1926.7	305.5	1821.5	298.1	1827.2	272.2	1837.2	287.8	1968.5	331.9

For a ranking for plan data, SO‐STD and MCO‐STD in first, SO‐SW600 in second, SO‐SW800 in third, SO‐SWnl and MCO‐SW in last.

## 4 DISCUSSION

The different methods offer clinically similar designs with some particularities. The *D*_98%_ and *D*_2%_ guidelines for the PTVs are met by all methods. Our findings show that the PTVs coverage is statistically most uniform with the SO. Moreover, SO gives statistically a most CI for the PTV‐56 Gy and a most TC. The trade‐off between the *D*_98%_ and a better likelihood of cure on the one hand, and the prescribed mean doses of the OARs nearby resulting to a possible lower local toxicity risk on the other, makes this type of integrated boost planning study particularly difficult. The study also showed that SO allows for dose reduction outside of the PTV‐56 Gy with the 50% isodoses of each of PTV. Despite being slight, the disparities nonetheless persist. The selection of the various approaches must consider all dosimetric variations between TC, dose reduction at OARs and the dose outside of the PTV‐56 Gy (healthy tissue). While the differences in PTV coverage between plans are frequently significant statistically, the authors question whether these comparatively small variations will have any therapeutic significance. The clinical implications for OARs are probably more important. Better dose distributions are probably going to lead to less toxicity because the mean dosage disparities between the different methods are relatively considerable for some OARs. This is true for both parotids, as well as the larynx and oral cavity for MCO‐STD where these OARs are significantly also relevantly more spared by MCO. With all methods, the average dose to the parotid glands is less than 26 Gy, thus reducing/limiting late xerostomia and helping to improve the quality of life of head and neck cancer patients with an additional benefit for the MCO.^3^, ^4^, ^5^ The MCO‐STD will allow better sparing of the larynx and oral cavity, which may decrease the risks of dysphagia and oral mucositis.^24^

A lower maximum dose to the spinal cord or brainstem may be advantageous because a proportion of patients will have a loco‐regional recurrence of their cancer: from 25% at 5 years to 40% at 10 years. There is also a significant advantage of MCO, it is potentially easier to re‐irradiate the patient in case of recurrence as the additional dose may be higher because the spinal cord or the brainstem were better spared during the first treatment.

This advantage of MCO on the reduction of doses to the OARs has already been studied since the first versions of RayStation.^8^, ^9^, ^10^, ^11^ Moreover, with its ability to navigate between the Pareto plans, MCO will offer the user the choice of finding the best compromise between the different OAR preservation objectives or rather the choice of treating the target volumes with the live visualization of the fluence result.^25^

Unfortunately, there is a small difference between the navigation plan and the final plan in dose (after conversion to deliverable machine parameters for each control point and the final dose calculated). For this reason, the segment‐based optimization using sliding sequencing to create a same plan after conversion in plan deliverable of the navigation plan. The system finds an identical plan between the fluence and the final plan deliverable by an accelerator. This is very interesting because in MCO‐STD, after conversion, it can happen that a clinical objective deteriorates, and the final plan is slightly worse than the desired navigation plan. In our study, however, we found that the plan resulting from MCO‐SW have a worse dosimetric result than MCO‐STD. But we are still in the early versions of this new mode and software updates have always bring improvements. For example, for MCO‐STD, the gap between the navigation plan and the deliverable plan has considerably decreased with the evolution of the software versions.

The calculation time of the Pareto database is much longer in MCO‐SW: approximately 10 min in MCO‐STD against approximately 2 h in MCO‐SW. Consequently, we consider that this extra time is not compatible for a clinical application and mainly the final result of the MCO‐SW plan is worse than the other plans.

In SO, the realization of the plan is different from the MCO. The user will modify the OARs’ weights or targets weights (in function of practice, in our institution, we only change the OARs weights) if the clinical goals are not reached, while trying not to degrade the coverage of the targets before restarting a novel optimization. This iterative scheme is performed several times to find the best solution and extremely subject to the experience of the user. This is made much smoother with the sliding windows. Indeed, it takes about half as many series of iterations to arrive at the final plan and the weights of the OARs are much less increased to arrive at the final solution. This is probably due to the high modulation of the MLC in SW and it is the major interest of SW that we found. The final plans have a similar dosimetric quality of SO‐STD. From a planning point of view SO‐SW shows its superiority to SO‐STD. Nevertheless, through to the navigation in the Pareto plans and to let the user choose to visualize the dose trade‐offs between the clinicals objectives, MCO‐STD shows itself superior for the realization of the plans.

For planning, to propose a ranking, MCO‐STD rank is first, SO‐SW ranks is second (the limitation of MU has no impact for the planification) SO‐STD ranks is third and MCO‐SW is the last.

Another difference between STD and SW mode lies in the physical aspect of the processing plan with the modulation of the MLC. In SW, the number of MUs is considerably increased and so is the complexity of the MLC with the increase of *M* and the decrease of MCSv. Due to the opening/closing sweep of the MLC shape, the aperture complexity *M* goes from a maximum (much higher than in STD MLC) to a minimum. The slots are more numerous, and the aperture complexity is higher for many control points when the number of MUs increase. By limiting the MUs, the sliding windows generated by the optimizer are less fine, which allows for improved GPR with the pretreatment QA. For the MCSv complexity, it is much lower in SW, which corresponds to a stronger modulation, mainly because of its AAV component value which is highly decreased. The AAV component corresponds to the opening of the MLC shape, which gives the same phenomenon as the *M* metric. The arcs are clearly more modulated with finer and smaller MLC shapes, which strongly increases the number of MUs. For SO‐SW600, the metric *M* have a similar value at SO‐STD plan where the number of MUs is close. For MCSv, to have a same value at SO/MCO‐STD, the maximum limit of MU must be 400 MUs. The values of the metrics follow the number of MUs, the more it is modulated, the more *M* increases and MCSv tends toward 0 its minimum value corresponding to a maximum modulation. This was the goal to highlight the difference of MLC in terms of field opening and complexity between STD and SW mode. The authors would like to warn against a maximum limit of MU that is too restrictive; at 400 MUs, SW are clearly less satisfactory: most of the clinical goals in Table 1 are no longer met.

VMAT plan complexity analysis is streamlines the VMAT planning process and can improve plan quality. While some highly modulated plans do pass pretreatment QA, measurement QA does not catch all clinically significant delivery errors, a high degree of modulation may not be required to meet planning goals.

Many institutions perform a pre‐processing measurement with gamma analysis followed the recommendations made by Miften et al.^17^ and if the plan does not meet the determined level, different actions must be carried out and potentially the plan can be invalidated for its delivery. This can generate an excess of activity for the services and the fact of choosing a more robust method in term of pre‐treatment and a less thorough modulation can be a good choice in order to guarantee a high level of quality of treatment for the patients by avoiding making treatment plans equivalent in dosimetric terms but whose correspondence between the measurement and the calculation is more perfectible. It is the choice in our institution.

The global gamma analysis shows no difference between the different methods. A more detailed analysis in local mode confirms the more advanced modulation with a decrease in the adequacy between calculation and measurement although the measurements remain satisfactory. Based on our experience with VMAT and pre‐treatment measurements, our study did not show any contraindication for treatment delivery even with high MUs. However, we will focus on limiting the number of MUs in a reasonable way. In our study, 800 MUs seems to be a good compromise between a dosimetric plan of the same quality as the SO‐STD referential plan, a satisfactory pre‐treatment measurement and a modulation controlled by the MLC complexity metrics. Previous studies^23^, ^26^ of complexity metric correlations generally report weak to moderate correlations to QA results. For this study, the complexity metrics selected were used to describe the general MLC movement and aperture shape of treatments, not to determine a correlation with the pre‐processing QA. The number of plans is low, 10 per method, not sufficient to establish a reliable correlation. Several factors influence the result of the pre‐processing QA as the detector and phantom used for measurement, the linac for delivery, the MLC leaf sequencing, the TPS used to realize the plans and particularly the accuracy of beam modeling. An energy, very well modeled, will allow a gain on the QA result. Thus, more complex plans can be delivered without degrading the QA result. However, this study can give interesting information's. The plans with a GPR with local normalization below 90%, that is, seven plans, have MCVs values below 0.113. Six plans have a value between 0.057 and 0.083, very low. For STD, the MCSv has a value around 0.172. The values in SW are among the lowest compared to those found in the literature.^23^, ^26^, ^27^, ^28^ While specific results may not be applied to other institutions, the results can be used to develop institute specific QA tools to aid in the treatment planning process. It seems judicious to use the complexity metrics to evaluate the modulation of leaf sequencing pattern. The MUs limitation parameter can be adjusted in order to have a desired complexity metric value or at least not exceeded in order to guarantee a better coherence between calculated dose and distributed dose. A future work, with more cases, could bring a more exhaustive conclusion on a possible correlation between complexity metrics and QA according to different MLC sequencings.

With a single arc in SW, the measurements are less good. However, for the two‐arc methods, if we look at the measurement/calculation correspondence arc by arc, we see a poorer match with GPR using the local 3%/3 mm criteria between 90% and 95% per arc. When we perform the pass rate with the two arcs, the pass rate is much better. This is because for Elekta accelerators and MLC optical leaf design, the random errors of the leaf between the two arcs are cumulated and thus a better result is obtained which may help explain a worse pre‐processing measurement in SW in addition to the different modulation.

In terms of treatment time, even though the SW treatment uses only one arc, the treatment time is extended by 50% (60 s more than the standard 125 s). The impact is more on patient comfort as the session is extended by 1 min. The intra‐fraction movement should not change much, as the patient is under a mask of contention, so it seems unlikely that this intra‐fraction movement will interfere with the treatment.

## 5 CONCLUSION

All methods evaluated offer satisfactory results and the plans are feasible for treatment. A clear advantage of the SW is that the treatment plan is simpler to produce it requires fewer changes in OARs weights and fewer iterations due to the more advanced modulation. However, care must be taken with the number of MUs before routine use otherwise there may be delivery problems to the accelerator and the plan may be invalidated by a low gamma pass rate. If the MCO is not adopted in clinical practice, the SO‐SW offers a serious alternative. The MCO stands out for its ease of use and will allow a less experienced user to offer a better plan than in SO. In addition, the MCO‐STD will reduce the dose to the OARs while maintaining good TC. Thus, MCO‐STD is proven superior yet for the head and neck VMAT treatment awaiting future improvements for the MCO‐SW.

## AUTHOR CONTRIBUTIONS

Julien Rolland realized the study. All contributing authors reviewed the manuscript and gave feedback on the findings.

## CONFLICT OF INTEREST STATEMENT

The authors declare no conflicts of interest.
